# Monitoring the Role of Physical Activity in Children with Flat Feet by Assessing Subtalar Flexibility and Plantar Arch Index

**DOI:** 10.3390/children9030427

**Published:** 2022-03-18

**Authors:** Ligia Rusu, Mihnea Ion Marin, Michi Mihail Geambesa, Mihai Robert Rusu

**Affiliations:** 1 Sport Medicine and Physical Therapy Department, Faculty of Physical Education and Sport, University of Craiova, 200585 Craiova, Romania; mihail.geambesa@edu.ucv.ro (M.M.G.); mihai.rusu@edu.ucv.ro (M.R.R.); 2 Industrial Engineering Department, Faculty of Mechanics, University of Craiova, 200585 Craiova, Romania; mihnea.marin@edu.ucv.ro

**Keywords:** flat foot, subtalar flexibility, plantar arch index, orthoses

## Abstract

Flat foot is a common pediatric foot deformity which involves subtalar flexibility; it can affect the plantar arch. This study analyzes the evolution of two parameters, i.e., plantar index arch and subtalar flexibility, before and after physiotherapy and orthoses interventions, and examines the correlation between these two parameters. Methods: The study included 30 participants (17 boys, 12 girls, average age 9.37 ± 1.42 years) with bilateral flat foot. We made two groups, each with 15 subjects. Assessments of the subtalar flexibility and plantar arch index used RSScan the platform, and were undertaken at two time points. Therapeutic interventions: Group 1—short foot exercises (SFE); Group 2—SFE and insoles. Statistical analyses included Student’s *t*-test, Cohen’s D coefficient, Pearson and Sperman correlation. Results: Group 1—subtalar flexibility decreased for the left and right feet by 28.6% and 15.9% respectively, indicating good evolution for the left foot. For both feet, a decrease of the plantar index arch was observed. Group 2—subtalar flexibility decreased for the right and left feet by 43.4% and 37.7% respectively, indicating a good evolution for the right foot. For both feet, a decrease of plantar index arch was observed. Between groups, subtalar flexibility evolved well for Group 2; this was attributed to mixt intervention, physical therapy and orthosis. For plantar arch index, differences were not significant between the two groups. We observed an inverse correlation between subtalar flexibility and plantar arch index. Conclusions: Improvement of plantar index arch in static and dynamic situations creates the premise of a good therapeutic intervention and increases foot balance and postural control. The parameter which showed the most beneficial influence was the evolution is subtalar flexibility.

## 1. Introduction

Flat foot (FF) describes flatness of the medial longitudinal arch (MLA) of the foot, which is visible in standing position but also in the gait; it may be categorized as either flexible or rigid. Hyperpronation or excessive pronation of the foot refers to ankle bones being turned inward and the rest of the foot turned outward. This happens when the arch of the foot is flattened when weight is applied to it. Both aspects open a discussion about the behavior of the subtalar angle which determines subtalar flexibility in functional activities [1].

Several discussions are also ongoing about the definition of subtalar angle and its relationship with foot pathologies.

Flat foot is a pathologies which often is associated with pain and instability. The condition requires an early diagnosis to determine its etiology and design therapeutic interventions.

The subtalar joint has two parts: anterior and posterior. Subtalar joint movements are inversion–eversion from a clinical point of view; the other components (e.g., anteroposterior and mediolateral translation) are not assessable by clinical examination [2]. The subtalar joint axis has an average inclination 42° in the sagittal plane and 23° medial deviation in relation to the long axis of the foot [3].

Discussions about this angle have stemmed from the different methods used for assessing its mobility; in this context, many authors agree that inversion is 25–30° and eversion 5–10°. In practice, the relevance of this angle seems not to be so important; as such, it is necessary to focus instead on subtalar flexibility. This flexibility is connected to the body mass index (BMI). In many cases, increased flexibility seems to be correlated with a lack of anterior facet of the joint which favorizes the development of flat foot [4].

Gait analysis is one method that could be used to design physiotherapy programs in patients with flat foot (FF) due to the need to understand the behavior of the foot during gait, which involves three actions: rollover of the foot from supination to pronation on the floor, dorsal and plantar flexibility, and rotations. Subtalar joint movement can be described as rotation, translation or a combination of both; motion of this joint can be broken into a rotation about, and a translation along, the helical axis. During weight-bearing motion, all of the bones in the foot rotate around the subtalar joint axis [5]. All of these afford the foot the capacity to adapt to different conditions whereby the foot absorbs the ground force effect.

In this context, the subtalar joint has an important role, i.e., it combines the inversion with internal rotation and the eversion with external rotation. The consequence of this is that it has a possible effect under the plantar arch and in cases of collapse of the arch and midfoot during inversion, but with the arch lifted during eversion.

Flat foot may be defined by the collapse of MLA and overpronation. The condition can cause pain, gait disorders, static and balance disorders and affect the entire lower limb [6,7].

Today, the early diagnosis of FF is a challenge because clinical practices have a lot of variations regarding evaluations and therapy of the condition. Most diagnoses are based on clinical evaluations and radiography rather than functional diagnoses, which assess the behavior of joints through static and dynamic movements. For this reason, many authors do not accept this diagnostic method [6].

Many evaluations include measuring indexes like the valgus index, arch index, Staheli index, Chippaux-Smirak index, Foot posture index and Clarke angle [8,9,10]. Many studies have been published on the differences in evaluation results depending of the index choice. As such, there is no consensus regarding the diagnosis of FF because the reliability of these measurements can be poor.

Some authors consider the Chippaux-Smirak to be the best solution for diagnoses of FF in children [11]. Chen et al. considers that this index is the gold standard in FF evaluations for children under 8 years of age [12].

In contrast, some authors consider that clinical examinations are the best approach [13]. Fascione et al. considered that there is a limit of concordance between the Clarke angle and Chippaux-Smirak index, with regard to the plantar arch status [14]. The same aspect is relevant for Langley, who considered that the Chippaux-Smirak index makes possible more accurate predictions and diagnoses than the Staheli index [15].

First evaluations of FF and physiotherapy approaches are often based on the plantar print foot and measurements of the indexes. Clinical examinations of the longitudinal arch give information about the probability of FF occurrence; however, medical equipment make it possible to analyze the gait using pressure and force plates, as well as also baropodography [16]. To determine the foot type, arch index values are used, based on the contact area of the middle section of the plantar footprint [16]; however, this has little relevance to clinical classifications of the foot.

A new approach to diagnosing and treating FF is the development of a cluster model which makes it possible to identify the etiology and assess the foot alignment, which can influence the foot dynamic during gait and determine how physical therapy could improve this [17,18].

The relationship between subtalar angle and MLA is the subject of debate, and some authors have written about the eversion of subtalar angle and flat foot arch and vice versa [19]. Based on this, hyperpronation is a feature of FF; this is just a hypothesis, because there are no conclusive studies to date, as numerous methods exist to measure the morphological parameters of the foot, and unanimity of which is most suitable has not been reached [20].

Menz et al. measured the correlation between the results of plantar arch measurements (using force and pressure plates) and radiographyc aspects of calcaneal angle inclination, as well as correlations between this angle and navicular bone position. They observed a strong correlation between plantar arch and calcaneal angle inclination [21].

Even if the majority of the researchers and physicians consider that approaches based on the plantar arch are the most appropriate for foot classifications, many acknowledge that there are inconsistencies regarding the indexes used for foot classifications, and that clinical assessments have shortcomings.

The literature reveals many questions regarding FF, in terms of clinical and functional diagnosis, biomechanic parameters, relationships between these parameters in normal foot and in FF, etc. Therapeutic interventions and orthesis require complex evaluations and follow up.

The hypothesis of this study is that biomechanical parameters (subtalar flexibility and index arch) are relevant for monitoring the evolution of FF and response to physiotherapy and orthoses interventions. The purpose of this study is to analyze the evolution of two parameters, i.e., plantar index arch and subtalar flexibility, before and after physiotherapy and orthoses interventions. Also, this study includes an analysis of the correlation between these two parameters.

## 2. Study Design

This study was conducted in the Sports Medicine Department of the University of Craiova. It conformed to the guidelines of the Declaration of Helsinki, and was approved by the Ethics Committee of University of Craiova (20–28 September 2021). Written informed consent was obtained from all participants. Clinical trial registration according to the ICMJE guidelines was registered with number 243/30.09.2021. 

A total of 30 participants with flat-foot were selected: 17 boys and 13 girls, with an average age of 9.37 ± 1.42 years. Both groups had bilateral flat foot degree II.

The inclusion criteria were bilateral flat foot diagnosed by clinical examination in standing position and during gait. The exclusion criteria were: (1) prior foot or ankle surgery; (2) pain in the lower extremities; (3) overweight or obese; (4) any other foot deformities; or (5) neuromuscular and neurological disorders.

We created two groups. Group 1 (15 subjects) included participants in the physiotherapy program. Group 2 (15 subjects) included participants in the physiotherapy program who also wear foot orthoses (insoles).

### 2.1. Evaluation

**Figure 1:**
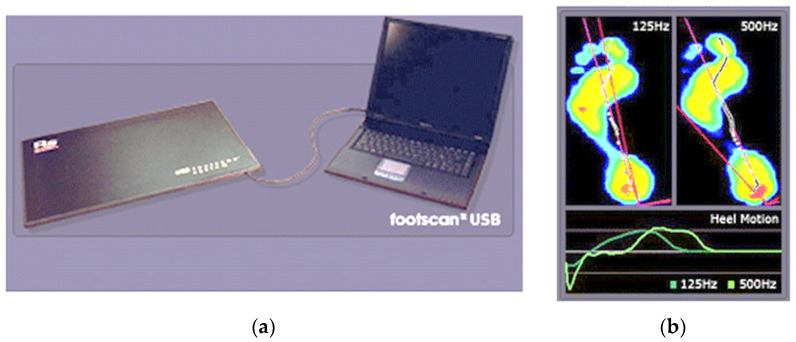
RSscan platform: (**a**) RSscan components; (**b**) RSscan software (Rsscan—RS software for footscan|Logemas).

Biomechanic evaluations consisted of assessing the subtalar flexibility and plantar arch index. For both parameters, we used a force and pressure platform RSScan (Figure 1).

Data were recorded in static and dynamic positions during a gait cycle. We made three measurements and then selected the best data, as it was necessary to obtain the entire plantar surface. We asked subjects to relax and walk with their normal gait.

Statistical analyses were performed using SPSS V.2.0 package.

The aim of the statistical analyses was to identify significant differences in subtalar flexibility and plantar index arch between the two groups of patients. We used student’s *t*-test, Cohen’s D coefficient, Pearson and Sperman correlation.

### 2.2. Intervention

Therapeutic interventions consisted of:

For group 1—The physiotherapy program included classic exercises, e.g.,:

- strengthening of ligaments;
- strengthening of the muscles of the shins and thighs, heel bones, metatarsal fingers, ankle joint and plantar aponeurosis;
- correction of the foot arch position;

The exercises included toe bending or towel-curl exercises which mobilize the extrinsic muscles of the foot, such as the flexor digitorum longus muscle [22].

Also, we proposed short foot exercises (SFE) which are a form of sensory-motor training that activates the intrinsic muscles in the foot and actively forms the longitudinal arch and the horizontal arch [23]. A posture was maintained for 10 s followed by 5 s of relaxing; this process was repeated for a total of 30 min per session, with three sessions per week for a total of 12 weeks. The exercises [24] included:

- Single leg stance on a fixed surface (three sets, 10 repetitions followed by a 10 s break)
- Forward lean on a fixed surface (three sets, 10 repetitions followed by a 10 s break )
- Standing on one leg on an unstable surface (three sets, 10 repetitions followed by a 10 s break)
- Forward lean on an unstable surface (three sets, 10 repetitions followed by a 10 s break)
- Throwing a ball with different directions on fixed surface (three sets, 10 repetitions followed by a 10 s break)
- Throwing a ball in different directions on an unstable surface (three sets, 10 repetitions followed by a 10 s break)
- Squat on a fixed surface (three sets, 10 repetitions followed by a 10 s break)
- Jump on a fixed surface (three sets, 10 repetitions followed by a 10 s break)
- Squat on an unstable surface (three sets, 10 repetitions followed by a 10 s break)
- Jump on an unstable surface (three sets, 10 repetitions).

For group 2—We proposed the same physiotherapy program but also recommended the use insoles to improve the medial longitudinal arch (MLA). In this way, we combined foot orthotics with sensory-motor training, i.e., SFE.

Foot orthosis reduces the severity of symptoms that are secondary to increased flexibility.

We prescribed personalized, semirigid insoles, manufactured by Ortoprotetica (Bucharest, Romania) based on 3D casting.

The orthoses were manufactured based on plantar pressure evaluations using the RSScan pressure platform. They featured a semirigid thermoplastic heel cup extending to the base of the metatarsals with a full-length, perforated ethylene vinyl acetate top cover. Each person had his/her own orthosis and was allowed to take part in normal activities. The insoles were given to the subjects, who were instructed to put them into their shoes.

**Figure 2:**
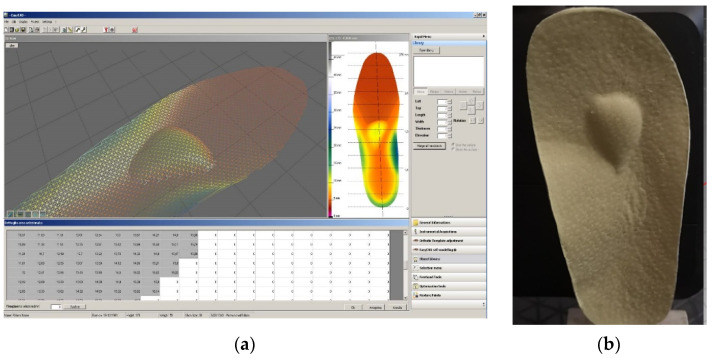
Insole: (**a**) CAD design of the insole; (**b**) Insole with arch support.

The insoles were designed based upon force-plate data using the CAD-CAM (CNC) method (Figure 2a,b).

In this way, we ensured arch supports in the insoles.

### 2.3. Statistical Analysis

The aim of the statistical analysis was to identify significant differences in subtalar flexibility and plantar arch index between the two groups of patients. Statistical analysis is also used to measure differences between first and second evaluations of these parameters for each group. This was intended to assess the evolution of each group and to measure the differences between groups 1 and 2 at the second evaluation.

Data analysis based software packages were used for statistical analyses.

A database was initially created containing the experimental data from the significant aspects of this research. The recording values of the parameters were analyzed to visualize the variables, and the statistical analysis was intended to reveal significant differences between the data series for each group. Descriptive data (means SD) were reported for the entire patient cohort. Normal distribution was tested using the JB test and visual analysis of Gauss function for both parameters. We applied a *t* test for equal and unequal variances depending on the results of the Levene test for all parameters. Statistical significance was set at *p* < 0.05.

All data presented herein have been normalized.

## 3. Results

The average anthropometric characteristics of the 30 participants were: weight 41.8 ± 12.72 kg, height 148.7 ± 10.96 cm, and body mass index 18.84 ± 5.32 kg/m^2^.

### 3.1. Evolution of Parameters between EV1 and EV2 for Each Group

We monitored changes in the aforementioned parameters before (EV1) and after (EV2) the physiotherapy program. The results are presented for each group, for right and left feet at EV1 and EV2.

**Figure 3:**
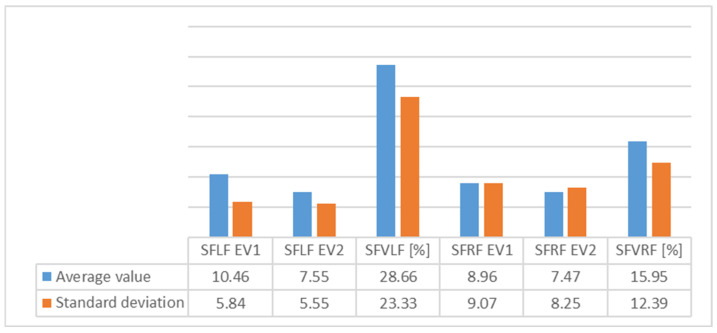
Variation of average values and standard deviation for subtalar flexibility for Group 1 between EV1 and EV2 (SFLF = subtalar flexibility for left foot; SFVLF = subtalar flexibility variation for left foot; SFRF = subtalar flexibility for right foot; SFVRF = subtalar flexibility variation for right foot).

Table 1 presents the evolution of subtalar flexibility and Figure 3 shows the average values of subtalar flexibility for group 1.

**Figure 4:**
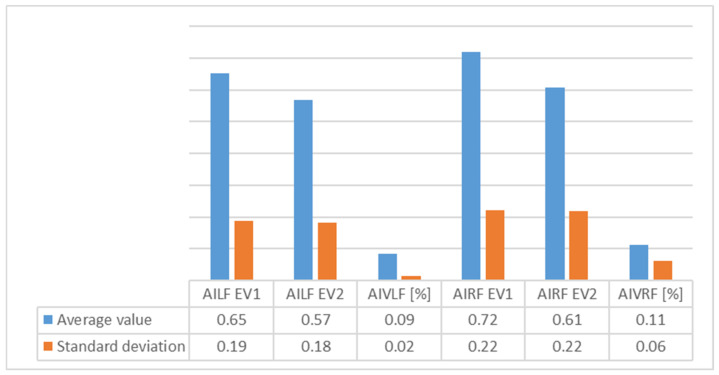
Variation of average values and standard deviation for plantar index arch for Group 1 between EV1 and EV2 (AILF = plantar index arch for left foot; AIVLF = plantar index arch variation for left foot; AIRF = plantar index arch for right foot; AIVRF = plantar index arch variation for right foot).

Table 2 presents the evolution of plantar index arch and Figure 4 shows the average values of plantar index arch for group 1.

**Figure 5:**
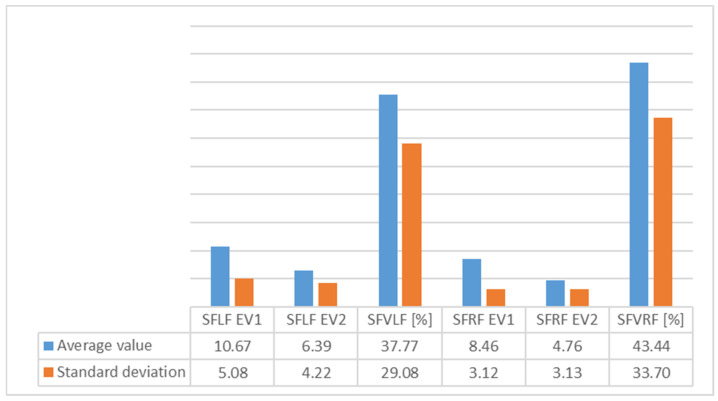
Variation of average values and standard deviation for subtalar flexibility for Group 2 between EV1 and EV2 (SFLF = subtalar flexibility for left foot; SFVLF = subtalar flexibility variation for left foot; SFRF = subtalar flexibility for right foot; SFVRF = subtalar flexibility variation for right foot).

Table 3 presents the evolution of subtalar flexibility and Figure 5 shows the average values of subtalar flexibility for Group 2.

**Figure 6:**
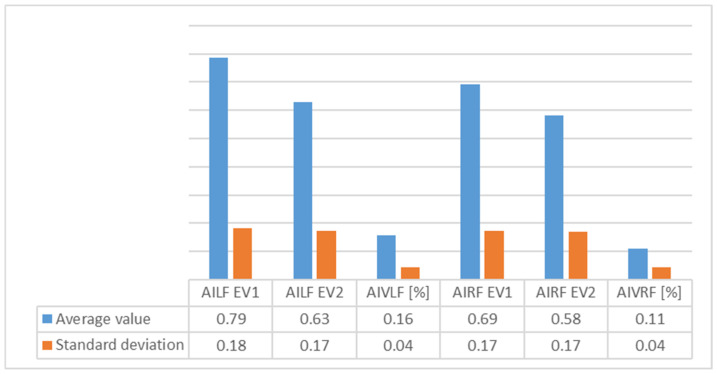
Variation of average values and standard deviation for plantar index arch for Group 2 between EV1 and EV2 (AILF = plantar index arch for left foot; AIVLF = plantar index arch variation for left foot; AIRF = plantar index arch for right foot; AIVRF = plantar index arch variation for right foot).

Table 4 presents the evolution of plantar index arch and Figure 6 shows the average values of index arch for Group 2.

### 3.2. Statistical Analysis of Parameters Evolution between EV1 and EV2, for Each Group

For both groups, the student’s *t*-test results and Cohen’s D coefficient in Table 5.

### 3.3. Statistical Analysis of Parameters, Comparison between the Two Groups

Student’s *t*-test results and Chen’s D coefficient are presented in Table 6.

### 3.4. Statistical Analysis of Parameter Variations, Comparison between the Two Groups

Student’s *t*-test results and Cohen’s D coefficient are presented in Table 7.

### 3.5. Statistical Correlation

Correlations between the two parameters were determined using two coefficients: Pearson and Spearman. The results are presented in Table 8.

## 4. Discussion

**Figure 7:**
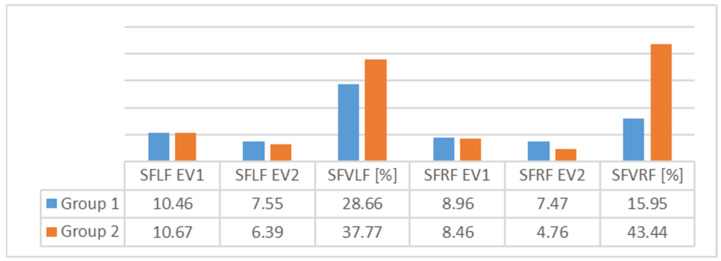
Average values for subtalar flexibility—comparison between the two groups (SFLF = subtalar flexibility for left foot; SFVLF = subtalar flexibility variation for left foot; SFRF = subtalar flexibility for right foot; SFVRF = subtalar flexibility variation for right foot).

**Figure 8:**
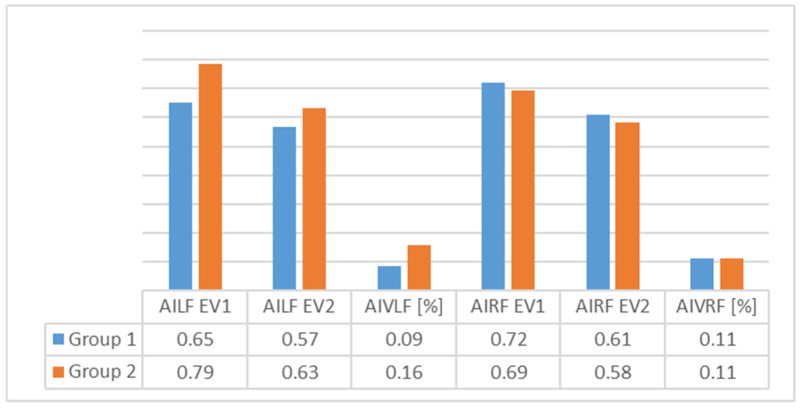
Average values for plantar index arch—comparison between the two groups (AILF = plantar index arch for left foot; AIVLF = plantar index arch variation for left foot; AIRF = plantar index arch for right foot; AIVRF = plantar index arch variation for right foot).

An analysis of Figure 3, Figure 4, Figure 5, Figure 6, Figure 7 and Figure 8 gives information about the evolution of each parameter for both groups.

For Group 1, we observed average values of subtalar flexibility (Figure 3) and noted an important decrease for the left (28.6%) and right feet (15.9%), indicating good evolution for the left foot.

The standard deviation (SD) showed homogeneity for both feet.

For Group 1, we observed in the average (Figure 4) arch index values that good evolution had occurred, as seen by decrease in the index (which, nonetheless, still remained outside out of normal values). We also observed a high decrease on the right foot, perhaps because of right dominance or because of the high degree of severity of FF in right foot among some of the subjects. The SD revealed more homogeneous values for the left foot.

In the average subtalar flexibility values (Figure 5) for Group 2, we observed an important decrease for the right (43.4%) and left feet (37.7%), indicating good evolution for the right foot. The SD showed homogeneity for both feet.

In the average arch index values (Figure 6) for Group 2, we saw that good evolution had occurred for both feet, as evidenced by decrease in the plantar index (which, nonetheless, still remained outside out of normal values). This decrease was more pronounced for the left foot than for the right, perhaps as a result of the physical therapy and orthoses interventions. SD showed homogeneity for both feet.

In Figure 7 and Figure 8, we can see differences in the average values of the two groups. The results indicate that the differences were greater for Group 2 for both feet.

We conclude that for Group 1, because *p* was more than 0.05, there was no significant difference between EV1 and EV2. For group 2, we observed a degree of high significant for subtalar flexibility and plantar index arch for both feet (Table 5).

In conclusion, the good evolution for both parameters in Group 2 was attributed to the mixed intervention, i.e., physical therapy and orthoses.

Table 6 reveals a highly significant difference regarding subtalar flexibility in the right foot (*p* = 0.001); also, for plantar index arch, the *p* value was significant value, indicating that at EV2, the values were not significantly different between the two groups.

In conclusion, we note that subtalar flexibility is the parameter which is most likely to influence positive therapeutic outcome.

Regarding the comparison of parameters between two groups, we observed a size effect, as assessed using the Cohen’s D coefficient at EV2; the result is presented in Table 6.

A comparison regarding the variations in the parameters revealed highly significant differences between two groups regarding subtalar flexibility for the right foot (*p* = 0.004) and plantar index arch for the left foot (*p* = 0.000) (Table 7).

For other data, *p* values greater than 0.05 indicated the lack of significant difference between two groups (Table 7).

In conclusion, we consider that that parameters to be take into consideration when monitoring the effects of physiotherapy and orthosis interventions ought to be subtalar flexibility and plantar index arch, as the evolution of both parameters showed significant differences between the two groups. A much greater difference was observed for the left foot for plantar index arch.

We observed that was an inverse correlation for the left foot for both groups, and for the right foot with Group 2 only. This means that a decrease of subtalar flexibility could generate an increase in plantar index arch, even if the values of the two coefficients are around 0.00. We conclude therefore that there is no correlation between these two parameters (Table 8).

As many authors have underlined, classifying flat foot is a complicated process. In this context, Menz et al. discussed possible ways to place the foot in one of three situations: hyperpronation, neutral position, supination [21,25]. In our research, we tried to create an algorithm with which to assess FF in a therapeutic context. The results of our research revealed that assessments of physiotherapeutic interventions (rehabilitation) including orthoses are need to continuously monitor the program protocols. This requirement is based upon many questions searches regarding measurement methods for flat foot assessments. Analyses of the relationship between subtalar angle and MLA have been made by many authors using radiographic methods. For example, Sinclair et al. evaluate this relationship during running and observed that it was not relevant, and that the subtalar angle is not a predictor of the development of FF. Despite this, the authors observed that after 45 min of running, significant changes of subtalar angle sometimes occurred [19]. These aspects justify our research, which involved static and dynamic conditions, and are also in accordance with our results regarding subtalar flexibility and its role in monitoring the evolution of FF during physical therapy.

Bosch et al. studied the correlation between radiographic measurements of calcaneal inclination angle and plantar index arch in children. They observed that the plantar arch normalizes at around 5 years age if therapeutic intervention occurs early on, due to its effect on the calcaneal inclination angle [26].

Regarding physical therapy interventions, Wong et al. outlined the lack of a real foot classification method that could be used for clinical and therapeutic decision-making according to scientific evidence. They proposed an analysis of two aspects: clinical evaluation results and plantar index arch (calculated using AutoCAD software from footprint photographs) in 11 children that were involved in a physical therapy program. The results demonstrated that the plantar index arch values derived from plantar footprint photographs showed excellent reliability in people with varying BMI. Foot-type classification may help clinicians and researchers to subdivide sample populations to better differentiate mobility, gait, or treatment effects among foot types [27]. This study was in accordance with our results, which highlights the importance of examining the literature on the morphofunctional parameters of FF before designing rehabilitation programs.

Our approach indicated that biomechanic evaluations of FF could be used to monitor the efficacy of physical therapy interventions. This is in according with Langley et al., who observed the absence of a consensus regarding the choice of a therapeutic approach based on the morpho-functional aspects of foot [15].

Halabachi et al. also described the potential value of a physiotherapeutic strategy based on the use of appropriate footwear, foot orthoses (shoe inserts), and physical therapy including stretching and strengthening [16,28]. They concluded that are a lot of shortcomings regarding complex assessments of FF involving correlations between foot biomechanical parameters. However, they made reference to a study by Evans et al. which recommended exercise for flat feet, in the form of barefoot walking [29]. The main focus of such an exercise program is on stretching tight structures, strengthening weak components and improving proprioception and postural balance, which is in line with the approach proposed in our study.

Our intention here is to improve the literature data, assist in the design of rehabilitation programs based on evaluations, and demonstrate that SFE causes the head of the metatarsal bone to approach the heel without bending the toes in a weight-bearing state where.

These aspects are more relevant, because surgery in FF is still controversial, and little information is available regarding the efficacy of conservative management of flexible flat foot in children [30].

The results of our research underline the importance of customized management in FF, in accordance with Kachoosangy et al., who suggested that management strategies should be customized to symptomatic patients or persons with hereditary gait disorders or other comorbidities which increase the probability of growing dysfunction over time [31].

Our results suggest that orthoses interventions have to be monitored and adaptable according to the evolution of two parameters studied in this research. In this context Evans et al. concluded that until such time that the parameters of FF have become standardized, the best approach is the use of available, evidence-based management models. Those authors support ongoing studies and conclude that physical exercises for foot flat and follow-up involving the use of modified footwear are effective [29].

The results of our research demonstrate that physiotherapeutic and orthoses interventions result in improved subtalar flexibility and plantar arch index due to their ability to reduce the compensatory role of muscle groups that promote inversion and increase the role of the long perionier muscle (PL), as noted by Cho et al. [32].

At the same time, Youn et al. analyzed the subtalar joint during and after orthoses intervention using radiography, and observed that custom-made rigid foot orthoses (RFO) in children older than 6 years of age with pes planus (flat foot) can bring about significant improvements in calcaneus-related radiographic indices, and subsequently, improve talus-related radiologic indices [33].

## 5. Conclusions

Analyses of two parameters, i.e., subtalar flexibility and plantar arch index, allowed us to design and monitor therapeutic interventions and evaluate the difference between physiotherapy intervention alone and combined methods.

Improvements of plantar index arch in static and dynamic situations created the premise of a good therapeutic intervention, resulting in increased foot balance and postural control.

There are a lot of studies on the effects of therapeutic footwear, wedges, and insoles, but none which examines the evolution of MLA.

Sensory-motor training such as SFE is insufficient; such approaches must be extended to include analyses of biomechnic FF parameters in different conditions.

Future studies are required to investigate the long-term results of combined treatment of SFE on postural balance in subjects with a flat feet, and to evaluate the efficacy of the combination of SFE and orthoses interventions based on biomechanic analyses of FF.

We found no correlation between subtalar flexibility and plantar arch index. However, perhaps an association between these two parameters in FF could be found by examining the anatomic changes in the subtalar joint and its impact under the dynamic and passive systems of the ankle–foot complex. Such a connection could give rise to improved therapeutic methods.

Future research is need to assess and design therapeutic programs for the treatment of flat foot. This study was limited by its small group of patients and the fact that the subjects did always adhere to the physical therapy program or use the insoles.

## Figures and Tables

**Table 1:** Evolution of subtalar flexibility (Group 1, *n* = 15).

Subject	Subtalar Flexibility, Left Side EV1	Subtalar Flexibility, Left Side EV2	Variation of Subtalar Flexibility, Left Side %	Subtalar Flexibility, Right Side EV1	Subtalar Flexibility, Right Side EV2	Variation of Subtalar Flexibility, Right Side %
P16	7.50	5.58	25.60	12.68	9.27	26.89
P17	20.79	13.03	37.33	17.82	15.78	11.45
P18	11.45	10.77	5.94	21.42	19.86	7.28
P19	2.79	1.87	32.97	4.57	3.98	12.91
P20	24.35	23.31	4.27	11.45	10.22	10.74
P21	8.74	7.70	11.90	18.64	16.10	13.63
P22	5.86	4.72	19.45	7.28	6.75	7.28
P23	10.19	6.04	40.73	9.30	8.15	12.37
P24	9.13	2.30	74.81	10.32	9.84	4.65
P25	6.05	5.67	6.28	−17.86	−16.48	7.73
P26	4.54	3.76	17.18	3.37	2.67	19.57
P27	14.86	12.27	17.43	12.83	10.35	19.33
P28	12.66	6.15	51.42	8.41	7.34	12.72
P29	8.57	7.73	9.80	8.88	3.94	55.63
P30	9.45	2.38	74.81	5.22	4.33	17.05

P1–P30 patient number; EV 1—evaluation 1, EV2—evaluation 2.

**Table 2:** Plantar index arch evolution (Group 1, *n* = 15).

Subject	Plantar Index Arch, Left Side EV1	Plantar Index Arch, Left Side EV2	Difference EV1–EV2	Plantar Index Arch, Right Side EV1	Plantar Index Arch, Right Side EV2	Difference EV1–EV2
P16	0.93	0.83	0.10	0.91	0.87	0.04
P17	0.51	0.41	0.10	0.57	0.45	0.12
P18	0.78	0.68	0.10	1.12	0.98	0.14
P19	0.73	0.63	0.10	0.95	0.86	0.09
P20	0.70	0.61	0.09	0.73	0.60	0.13
P21	0.79	0.7	0.09	0.73	0.62	0.11
P22	0.80	0.71	0.09	0.79	0.72	0.07
P23	0.39	0.30	0.09	0.32	0.29	0.03
P24	0.41	0.32	0.09	0.48	0.33	0.15
P25	0.48	0.39	0.09	0.58	0.42	0.16
P26	0.58	0.50	0.08	0.62	0.55	0.07
P27	0.76	0.68	0.08	0.66	0.56	0.10
P28	0.78	0.70	0.08	1.05	0.78	0.27
P29	0.84	0.77	0.07	0.81	0.78	0.03
P30	0.32	0.28	0.04	0.48	0.32	0.16

P1–P30 patient number; EV 1—evaluation 1, EV2—evaluation 2.

**Table 3:** Evolution of subtalar flexibility (Group 2, *n* = 15).

Subject	Subtalar Flexibility, Left Side EV1	Subtalar Flexibility, Left Side EV2	Variation of Subtalar Flexibility, Left Side %	Subtalar Flexibility, Right Side EV1	Subtalar Flexibility, Right Side EV2	Variation of Subtalar Flexibility, Right Side %
P1	3.78	2.23	41.01	7.87	5.72	27.32
P2	14.32	0.76	94.69	6.85	2.39	65.11
P3	14.73	12.36	16.09	3.51	1.52	56.70
P4	12.43	11.57	6.92	11.37	9.28	18.38
P5	7.64	1.95	74.48	4.88	4.67	4.30
P6	8.61	2.42	71.89	9.21	3.54	61.56
P7	15.34	13.85	9.71	8.77	7.32	16.53
P8	8.53	7.21	15.47	5.05	4.87	3.56
P9	4.70	4.64	1.28	5.74	−1.28	122.30
P10	15.29	8.65	43.43	10.33	6.83	33.88
P11	16.61	6.08	63.40	7.88	3.65	53.68
P12	7.49	6.37	14.95	12.08	0.25	97.93
P13	10.64	8.91	16.26	15.56	9.25	40.55
P14	1.66	0.99	40.36	8.67	5.87	32.30
P15	18.26	7.91	56.68	9.12	7.53	17.43

P1–P15 patient number; EV 1—evaluation 1, EV2—evaluation 2.

**Table 4:** Plantar index arch evolution (Group 2, *n* = 15).

Subject	Plantar Index Arch Left Side EV1	Plantar Index Arch Left Side EV2	Difference EV1–EV2	Plantar Index Arch Right Side EV1	Plantar Index Arch Right Side EV2	Difference EV1–EV2
P1	0.79	0.54	0.25	0.68	0.55	0.13
P2	0.87	0.64	0.23	0.81	0.71	0.10
P3	0.94	0.74	0.20	0.66	0.58	0.08
P4	0.57	0.38	0.19	0.51	0.39	0.12
P5	0.96	0.78	0.18	0.43	0.32	0.11
P6	0.79	0.62	0.17	0.84	0.79	0.05
P7	1.04	0.89	0.15	0.80	0.75	0.05
P8	0.95	0.80	0.15	0.95	0.81	0.14
P9	1.00	0.87	0.13	0.85	0.74	0.11
P10	0.68	0.56	0.12	0.57	0.44	0.13
P11	0.70	0.58	0.12	0.87	0.65	0.22
P12	0.85	0.73	0.12	0.76	0.66	0.10
P13	0.65	0.53	0.12	0.61	0.49	0.12
P14	0.64	0.53	0.11	0.69	0.54	0.15
P15	0.38	0.27	0.11	0.35	0.29	0.06

P1–P15 patient number; EV 1—evaluation 1, EV2—evaluation 2.

**Table 5:** *p* value (student’s *t*-test) * and Cohen’s D coefficient ** comparing the results between EV1 and EV2.

Group	Tests	Foot Side	Subtalar Flexibility	Plantar Index Arch
Group 1	student’s *t*-test	right	0.321	0.088
	student’s *t*-test	left	0.086	0.110
	Cohen’s D coeff.	right	0.176	0.522
	Cohen’s D coeff.	left	0.528	0.472
Group 2	student’s *t*-test	right	0.001	0.042
	student’s *t*-test	left	0.009	0.012
	Cohen’s D coeff.	right	1.225	0.677
	Cohen’s D coeff.	left	0.947	0.900

significance level *p* < 0.05. 0.2 ≤ Cohen ≤ 0.5 small size effect; 0.5 ≤ Cohen ≤ 0.8 medium size effect; Cohen ≥ 0.8 high size effect.

**Table 6:** *p* value (student’s *t*-test) * and Cohen’s D coefficient ** for parameters at EV2 comparing the results of the two groups.

Tests	Foot Side	Subtalar Flexibility	Plantar Index Arch
student’s *t*-test	right	0.001	0.348
student’s *t*-test	left	0.262	0.170
Cohen’s D coeff.	right	0.449	2.198
Cohen’s D coeff.	left	0.243	0.365

significance level *p* < 0.05. 0.2 ≤ Cohen ≤ 0.5 small size effect; 0.5 ≤ Cohen ≤ 0.8 medium size effect; Cohen ≥ 0.8 high size effect.

**Table 7:** *p* value (student’s *t*-test) * and Cohen’s D coefficient ** for parameters variations at EV2 comparing tje two groups.

Tests	Foot Side	Subtalar Flexibility	Plantar Index Arch
student’s *t*-test	right	0.004	0.495
student’s *t*-test	left	0.175	0.000
Cohen’s D coeff.	right	1.120	0.003
Cohen’s D coeff.	left	0.357	0.148

significance level *p* < 0.05. 0.2 ≤ Cohen ≤ 0.5 small size effect; 0.5 ≤ Cohen ≤ 0.8 medium size effect; Cohen ≥ 0.8 high size effect.

**Table 8:** Person and Sperman correlation coefficients.

Groups	Parameter	Pearson Coefficient	Spearman Coefficient
Group 1	EV1 subtalar flexibility/plantar index arch left foot	−0.04	0.19
Group 1	EV2 subtalar flexibility/plantar index arch left foot	0.22	0.23
Group 1	EV1 subtalar flexibility/plantar index arch right foot	0.27	0.14
Group 1	EV2 subtalar flexibility/plantar index arch right foot	0.27	0.16
Group 2	EV1 subtalar flexibility/plantar index arch left foot	−0.26	−0.16
Group 2	EV2 subtalar flexibility/plantar index arch left foot	0.03	0.017
Group 2	EV1 subtalar flexibility/plantar index arch right foot	−0.22	−0.26
Group 2	EV2 subtalar flexibility/plantar index arch right foot	−0.53	−0.54

EV 1—evaluation1; EV 2—evaluation 2.

## Data Availability

Not applicable.
